# A word-of-mouth perspective on consumers of family medicine services: a case study

**DOI:** 10.25122/jml-2022-0098

**Published:** 2022-05

**Authors:** Traian Soare, Ciprian Ianovici, Iuliana-Raluca Gheorghe, Victor Lorin Purcărea, Cristina Maria Soare

**Affiliations:** 1. Department of Healthcare Marketing and Medical Technology, Carol Davila University of Medicine and Pharmacy, Bucharest, Romania; 2. Department of Oncological Surgery, Oncological Institute Prof. Dr. Al. Trestioreanu, Bucharest, Romania

**Keywords:** family physician, word-of-mouth, health care services, marketing healthcare services, WOM – word-of-mouth.

## Abstract

In an increasingly competitive health care market, family physicians have to elaborate and implement new strategies to attract potential patients. A useful and powerful method is word-of-mouth (WOM) because it shapes the consumers' attitudes and behaviours. Based on the recommendations of actual consumers, potential health care patients choose their family physicians. The aim of this study was to investigate the usefulness of WOM in family medicine and determine the key factors in recommending a certain family physician. The sample consisted of 338 patients under the supervision of a family physician, and the instrument for collecting data was a self-administered questionnaire. The findings revealed that the most important factors in spreading WOM are the communication skills and the expertise of the family physician. In addition, for patients between 27–33 years and 41–47 years, expertise is an absolute skill, whereas, for the health care consumers between 21 and 26 years, communication skills are essential in spreading WOM. Further, WOM becomes relevant in the family physician's activity as it may contribute to the delivery of value and in building sustainable physician-patient relationships.

## INTRODUCTION

For the Romanian health care system, a family physician is the gatekeeper of care, being the first contact point with open and unlimited access to its users [1]. Usually, the family physician's activity is person-centered, namely towards the beneficiary of the services, their family, and the community. The main scope of a family physician's activity is to provide a unique consultation process established on a long-term period of trust, by using effective communication strategies. Broadly, a family physician is responsible for providing comprehensive and continuing care for individuals, regardless of age, gender or illness. In a specific approach, family medicine activities concentrate on health and prevention or palliation services.

Since Romania has one of the strongest primary care structures [2], this has introduced a need for competition and the freedom of patient choice for a family physician. For instance, Romania is an example of a country where hospital spending has increased for illnesses that may be preventable and treatable in a timely and efficient manner by a family physician [3]. As such, starting with 2019, guided by the National Health Strategy 2014–2020, Romania is in a continuous process of elaborating and implementing a series of primary health care reforms that aim to strengthen the population's access, quality, and efficiency to the family medicine services.

An efficient family medicine service relates to patients' perceived health care quality, being associated with continuity, coordination, comprehensiveness of care, and community orientation [4]. In practice, the core elements associated with a family medicine activity have a common particularity, the physician-patient relationship or the patient experience.

Doyle explained that a patient experience comprises two types of experiences: rational and functional [5]. The rational experience refers to the interpersonal aspects of care, namely the physician's ability to treat patients with respect, compassion, and empathy, as well as their encouragement of patients' engagement behaviour in their decision-making process. As such, patients may get empowered with information regarding their health and expectations, but during this process, an entropy of information is formed because consumers will determine their perceived quality of the services received based on their expectations, be they emotional rather than the real experiences, the rational approach.

This empowerment with information may come from the family physicians but may also be provided through word-of-mouth.

Word-of-mouth is a very powerful instrument used in medicine that plays an essential role in influencing consumer expectations [6], pre-usage attitudes and expectations [7], as well as the post-usage perceptions of a service [8]. In family medicine, WOM may prove to be of great help in choosing the family physician, mostly in consumer decision-making. Moreover, WOM is an indicator of providing value in a patient experience and may ensure a sustainable relationship.

In the context of health care services, there is limited research conducted on WOM, and studies focused on this topic addressed this concept in general terms without selecting a particular medical specialty. However, a research study by Leisen and Hyman focused on primary care and WOM [9]. This study emphasized the key factors of WOM: the importance of building a physician-patient relationship and trust. Although the vast literature about WOM identified more factors in spreading WOM, more empirical research needs to focus on health care and be conducted in this field.

Thus, this study aimed to investigate the usefulness of WOM in family medicine and determine the key factors in recommending a certain family physician.

### The concept of WOM in health care services

Frequently, health care consumers talk to other consumers about their patient experiences in the form of communication called word-of-mouth (WOM) [10]. According to Arndt, WOM is an “oral, person-to-person communication between a receiver and a communicator between whom the receiver perceives as non-commercial” [11]. The interactivity and the spread of WOM make it a very effective source of information, especially for services in a pre-purchase experience stage [12].

Moreover, WOM becomes essential for services that are complex and are perceived as high risk [13], such as health care [14]. According to Lim and Chung, WOM is especially important in health care services due to its heterogeneity, the higher risks associated, and intangible nature [15]. In practice, in order to make the best health decision, consumers use WOM referrals for reassurance or confirmation that they made the right decision, lowering the risks related to health care services [16]. Further, many people rely on WOM recommendations about physicians from family members and friends or peers [17].

A deeper understanding of the factors related to WOM, for instance, reduces the unnecessary change of physicians, the retaking of several medical investigations, and asking for a second opinion. These actions have several consequences, but the positive one worth mentioning is the reduction of health costs [18]. Even though patients nowadays benefit from more information, we assume they are more educated and have improved their access to various online sources compared to what happened 10 years ago, but seeking health information remains a complex process [19].

In a physician-patient relationship, the patient may get health care information from informal sources, such as personal experience, referrals, public reporting, and recommendations, and from formal sources, the actual providers of the health care services [20].

In an increasingly competitive market, such as family medicine, the health care providers have to seek new methods to achieve advantages offered by WOM, as it shapes the consumers' attitudes and behaviours [21]. More precisely, WOM has a direct impact on the selection of a health care provider, and compliance with treatment is considered a significant measure of patient satisfaction [22]. These are the main reasons we need to determine the dimensions of WOM in health care services [23].

### The dimensions of WOM in health care services

According to Argan [24], there are four dimensions of WOM in health care services. We believe that not all of them are typically encountered in a family practice office. So, the most important dimensions that lead to the high spread of WOM are the communication skills and the expertise of the family physician.

The vast body of literature regarding the communication skills of physicians in health care services has linked them to the adherence to the treatment of patients, as well as to their satisfaction [24]. High physician communication skills such as listening, coaching, questioning, and explaining contribute significantly to WOM [25]. For instance, a physician gets to know the patient's health issues better and offers the possibility of creating a therapeutic relationship [25] by also referring to the emotion and central components [26]. We may conclude that communication skills lead to relationship-centered care and are an integral component of the communication process and the outcome of a physician's care [27]. In this context, investigating the key communication factors may uncover interesting aspects.

Expertise is associated with the professional experience of a physician. However, for some persons, expertise means that the source of information, in this case the physician, has knowledge, experience, and skills about a specific subject and may successfully diagnose and treat patients [28]. We have already acknowledged that the majority of patients prefer physicians who have excellent medical skills [29], but they usually conclude that a physician has expertise based on their post-consultation experiences in terms of how successful the treatment was. Some patients consider that the professional level is more important than the communication skills [30] and are more likely to express their opinions to family, friends, and peers about their experiences, the medical success, and the expert status of a physician.

The objectives of this research are twofold: (i) to investigate WOM in a family medicine office, and (ii) to determine the key factors of communication skills and expertise in their role in spreading WOM.

## MATERIAL AND METHODS

The research was conducted in a family physician's office for 3 months (March–May 2020). The sample consisted of 338 patients under the supervision of the family physician. The instrument for collecting data was a self-administered questionnaire that consisted of two sections:

- The first section concentrated on gathering demographic information about the respondents, such as gender and age;
- The second section focused on collecting information about WOM and its two dimensions: communication skills and expertise.

The data were analysed with IBM SPSS Statistics 20. The quantitative variables were tested for normal distribution with the Shapiro-Wilk test, and means, standard deviations, and interpercentile intervals were used, whereas the categorical data were depicted with the help of percentages and frequencies. In order to determine the differences between samples, the Chi-square test was used for the qualitative variables.

## RESULTS

The sample consisted of 221 women (65.4%) and 117 men (34.6%), with the ages between 27–33 years (24%), followed by respondents with ages between 34–40 years (13%) and 48–54 years (13%).

Regarding the medical history, the vast majority of participants suffered from cardiac disorders and diseases (46.9%), followed by cancer, mostly in the women's sample (27.3%), and other pathologies, such as diabetes mellitus (23.4%). Moreover, 32.2% of the respondents suffered from chronic diseases, which included cardiology (53.20%), diabetes (18.30%), and neurology (10.10%).

When choosing a family physician, 3.8% of the respondents had difficulties because the location of the family practice office was too far from their homes (53.8%), or they felt an incompatibility between them and the physicians (46.2%).

**Figure 1:**
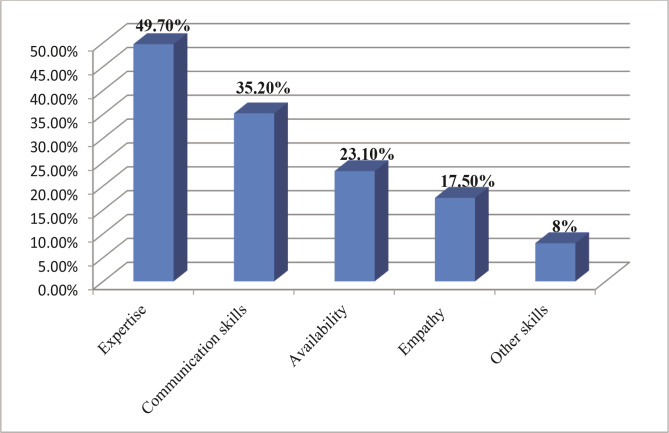
The dimensions of WOM in family medicine.

When mentioning the skills a physician must have, most respondents placed expertise and communication skills as being the most important ones (49.70% and 35.20%), followed by availability (23.10%) (Figure 1).

Further, most participants visited their family physician regularly (60.4%) or every six months (18.6%). Although most respondents do not regularly go for a consultation, they feel they have built a strong relationship with their family physician (85.5%).

Regarding the expertise dimension of WOM, we measured it through the perceived quality of the family physician services and by using other specific elements of the patient experience. As such, most of the respondents implied that they were pleased (34.9%) and very pleased (63.6%) with the quality of the family medicine services provided. In addition, the participants also mentioned being pleased and very pleased (37% and 64.5%) with the consultation procedure performed by the family physician. Even if some of the patients were not pleased with the prescribed treatment (1.4%), the vast majority were pleased (39.1%) and very pleased (59.5%) with the prescribed treatment by the family physician. Further, the expertise of the family physician was investigated by the explanation level provided to the patients. Most of the patients mentioned that the family physician explained the analysis in great detail (57.4%).

The statistically significant differences between the subsamples depending on the age of the respondents and the importance of the perceived expertise of the family physician uncovered that patients with ages between 27–33 years (30.4% *vs*. 17.6%) and 41–47 years (14.9% *vs*. 7.6%) believed that expertise is an absolute skill for a family physician, while the participants with ages between 62–68 years (11.8% *vs*. 2.4%) and over 75 years (9.4% *vs*. 1.8%) did not find the usefulness of expertise (p<0.001). (Table 1)

**Table 1:** The distribution of the patients depending on their age and the importance of the family physician's expertise.

Age/Expertise	Not useful		Useful		P-value*
	Frequency	Percentage	Frequency	Percentage	
**<20 years**	2	1.2%	0	0%	<0.001
**20–26 years**	6	3.5%	12	7.1%	
**27–33 years**	30	17.6%	51	30.4%	
**34–40 years**	20	11.8%	24	14.3%	
**41–47 years**	13	7.6%	25	14.9%	
**48–54 years**	28	16.5%	16	9.5%	
**55–61 years**	18	10.6%	23	13.7%	
**62–68 years**	20	11.8%	4	2.4%	
**69–75 years**	17	10%	10	6%	
**>75 years**	16	9.4%	3	1.8%	

* – Pearson Chi-Square Test.

Regarding the communication skills, most participants were satisfied (40.5%) and very satisfied (52.4%) with the fact that the family physician answered their questions by showing a significant interest in solving the patients' health issues (55.3%). Nevertheless, most respondents stated that they were engaged in the decision-making process related to their health and were informed accordingly (97.6%) by their family physician. Also, the family physician provided information about the diagnosis procedure (47.6%).

Further, the patients between 20–26 years concluded that communication skills are very important in the family physician activity (9.2% *vs*. 3.2%, p=0.012). (Table 2)

**Table 2:** The distribution of the patients depending on their age and the importance of the family physician's communication skills.

Age/Communication skills	Not useful		Useful		P-value*
	Frequency	Percentage	Frequency	Percentage	
**<20 years**	0	0%	2	1.7%	0.012
**20–26 years**	7	3.2%	11	9.2%	
**27–33 years**	50	22.8%	31	26.1%	
**34–40 years**	24	11%	20	16.8%	
**41–47 years**	26	11.9%	12	10.1%	
**48–54 years**	26	11.9%	18	15.1%	
**55–61 years**	31	14.2%	10	8.4%	
**62–68 years**	17	7.8%	7	5.9%	
**69–75 years**	22	10%	5	4.2%	
**>75 years**	16	7.3%	3	2.5%	

* – Pearson Chi-Square Test.

**Figure 2:**
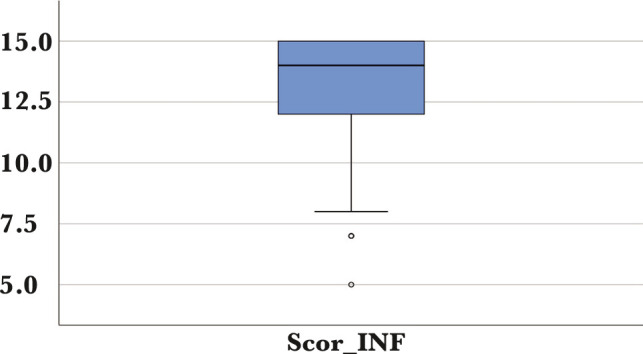
The distribution of the mean score of the perceived patient satisfaction regarding the communication skills of the family physician.

A mean score was calculated to determine the satisfaction of patients regarding the communication skills of the family physician. This outcome revealed that most patients were very satisfied with the communication skills of the family physician (Table 3 and Figure 2).

**Table 3:** The mean score of the perceived satisfaction of patients regarding the communication skills of the family physician.

Parameter	Mean (SD)	Median (IQR)	Min	Max
**Communication skills score**	13.27 (1.81)	14 (12–15)	5	15

The measurement of the perceived satisfaction with the services provided by the family physician suggested that most patients felt pleased (43.8%) and very pleased (54.4%) with the consultations. It was very useful to discover this because the body of literature on WOM pointed out that the importance of satisfaction is becoming a strategic direction of spreading WOM. Therefore, we may assume that the actual patients may become opinion leaders and, based on the perceived satisfaction, influence the decision of potential patients when choosing a family physician.

Moreover, the WOM attitude may be shaped by perceived improvement changes in the delivery of the family medicine services. In our case, 25.1% of patients stated that some improvements need to be conducted when delivering the service, in terms of waiting time (35.3%), appointments (24.7%), and the availability of the family physician (20%). Some of the patients even mentioned as an improvement integrating online communication into the family physician's activity (12.9%).

The essence of WOM is recommending, so the vast majority of the patients will use referrals for the services provided by the family physician (97.9%) to their friends, family, and other peers. In fact, the actual patients used recommendations of other patients (84%) when searching for their family physician and the recommendations of other physicians (12.4%).

## DISCUSSION

Health care services are often very difficult to be judged by patients because of the entropy of information. Although many patients have a growing interest in getting engaged in the decision-making process, their comprehensive perspective of the health care information is restrained to the functional aspects of a service [31]. Such information might be obtained by using WOM in an online or offline format and may reveal authentic information provided by other healthcare consumers who have already experienced a health care service offered by a specific physician and followed the medical treatment [32].

More than 40% of the scientific articles found in the literature about WOM and health care services emphasize that this type of communication process has an informal particularity, and most of the messages describe the post-consumption or post-purchase behaviour [33]. However, articles about the factors contributing to the spread of WOM in health care services are limited. Some specialists consider that the components of the health care factors are the interpersonal ones, starting with the communication process, the behaviour of the physicians and the medical staff, and ending with the expertise and skills of the physician, in terms of courtesy, respect, ability to actively listen and empathy [34], and, the atmospheric ones associated with the ambience, cleanliness, meals, cafeteria, accommodation, and other background factors [35].

WOM is a powerful form of marketing that is easily used by health care specialists, even in family medicine. The reasons to apply the principles of marketing and harness the outcomes of WOM in family medicine are based on the usefulness of quick, convenient, and free recommendations, as well as helpful in building awareness, prevention, and contributing to the education of potential consumers. Moreover, WOM helps the family physician deliver value and build sustainable relationships with their patients.

According to our study, the two dimensions of WOM that contributed efficiently to the dissemination of health care information are the perceived expertise and the communication skills of the family physician. The findings revealed a statistically significant difference in perceived expertise; namely, respondents between 27–33 and 41–47 years strongly believe that expertise is an absolute skill for a family physician compared to participants between 62–68 or over 75 years. The literature on WOM argues that persons over 65 are more likely to spread WOM about health care services to their family and friends [36]. Therefore, we may conclude that for this particular age group, the communication skills of the family physician are more important than his expertise.

The communication skills of a family physician are also important for younger patients between the ages of 21 and 26 years. This outcome becomes relevant in health care, where the use of information sources such as family and friends decreases with the increase of age [33].

No matter the reasons for spreading WOM, a further direction of research may be the impact of the emotional support offered by the family physician and whether this influences the spread of WOM [37].

A very interesting approach would also resume to the family physician building his online patient community for his actual and potential patients.

The limitations of the study are connected to the generalizability of the results, this being a disadvantage of the cross-sectional studies, the sample size, and the diversity in the target sample, which may include other patients of other family physicians. Lastly, the research was conducted in Bucharest, and it might be interesting to aim for rural areas of Romania as well.

## CONCLUSION

WOM is a very powerful instrument in health care services, especially in family medicine, where the level of competition increases day by day. WOM is determined by satisfied health care consumers and is spread from actual patients to potential patients. The two dimensions that may trigger effective WOM are the expertise and the communication skills of the family physician. By using WOM, a family physician builds a reputation and also a sustainable relationship with his actual health care consumers.
